# Patient-Reported Benefits and Limitations of Mobile Health Technologies for Diabetes in Pregnancy: Protocol for a Scoping Review

**DOI:** 10.2196/29727

**Published:** 2021-10-29

**Authors:** Katelyn Sushko, Qi Rui Wang, Holly Tschirhart Menezes, Donna Fitzpatrick-Lewis, Diana Sherifali

**Affiliations:** 1 School of Nursing Faculty of Health Sciences McMaster University Hamilton, ON Canada; 2 Diabetes Care and Research Program Hamilton Health Sciences Hamilton, ON Canada

**Keywords:** diabetes, pregnancy, type 1 diabetes, type 2 diabetes, gestational diabetes mellitus, mobile health, mHealth, virtual care, scoping review

## Abstract

**Background:**

For women with pre-existing and gestational diabetes mellitus, pregnancy involves specialized and intensive medical care to improve maternal and infant outcomes. Medical management for patients with diabetes in pregnancy typically occurs via frequent face-to-face outpatient appointments. Barriers to face-to-face care during the COVID-19 pandemic have signaled the need for high-quality, patient-centered virtual health care modalities, such as mobile health (mHealth).

**Objective:**

The objective of the proposed scoping review is to identify the patient-reported benefits and limitations of mHealth technology among women with diabetes in pregnancy. We also aim to determine how the women’s experiences align with the best practice standards for patient-centered communication.

**Methods:**

Arksey and O’Malley’s framework for conducting scoping reviews with refinements by Levac et al will be used to guide the conduct of this scoping review. Relevant studies will be identified through comprehensive database searches of MEDLINE, Embase, Emcare, and PsycINFO. Following database searches, studies will be screened for eligibility at the title, abstract, and full-text level by two independent reviewers, with the inclusion of a third reviewer if required to reach consensus. Data charting of included studies will be conducted by one reviewer using a standardized data extraction form and verified independently by a second reviewer. Synthesis of results will be guided by Thomas and Harden’s “Methods for the Thematic Synthesis of Qualitative Research in Systematic Reviews.”

**Results:**

As of August 2020, we have carried out the qualitative searches in the electronic databases MEDLINE, Embase, Emcare, and PsycINFO (Ovid interface) for a combined total of 8207 articles. Next, we plan to conduct the quantitative searches in the electronic databases MEDLINE, Embase, and Emcare (Ovid interface). We also plan to review the reference lists of relevant studies to identify additional eligible studies.

**Conclusions:**

With the results of this review, we hope to describe the patient-reported benefits and limitations of mHealth technology for women with diabetes in pregnancy. Furthermore, we aim to determine how women’s experiences align with the best practice standards for patient-centered communication. Ultimately, our review can provide valuable information for guideline developers, policy makers, and clinicians related to mobile technologies to support virtual care delivery for women with diabetes in pregnancy.

**International Registered Report Identifier (IRRID):**

PRR1-10.2196/29727

## Introduction

Diabetes is estimated to affect 20.4 million births or 15.8% of pregnancies worldwide [1]. Of these, 83% of cases are attributed to gestational diabetes mellitus, with the remaining 17% due to type 1 and type 2 diabetes [1]. It is well-established that diabetes in pregnancy is associated with a significant risk of adverse pregnancy outcomes [1-4]. These include an increased risk of congenital anomalies, stillbirths, and infant death among pregnancies complicated by gestational and pre-existing diabetes compared to the background population [1-4]. There is also a high occurrence of premature delivery, birth injuries, need for neonatal intensive care, and maternal pre-eclampsia, as well as other complications among pregnancies affected by diabetes [1-4].

For women with both gestational and pre-existing diabetes, there is a strong inverse relationship between maternal glycemic control and adverse pregnancy outcomes [5,6]. A large multicountry study that included over 25,000 participants with gestational diabetes found a 5-fold increased risk of macrosomia among infants of mothers with fasting plasma glucose of 5.6-5.8 mmol/L compared to infants of mothers with fasting plasma glucose less than 4.2 mmol/L [5]. Among pregnant women with pre-existing diabetes, a systematic review found that, on average, there was a 3-fold increased risk of congenital anomalies, miscarriage, and perinatal mortality among expectant mothers with poor glycemic control compared to those with good glycemic control [6]. Additional studies have reported similar findings, strengthening the link between glycemic control during pregnancy and maternal and infant outcomes [7-11].

As the evidence indicates that improved glycemic control during pregnancy optimizes perinatal outcomes, expectant mothers with diabetes receive intensive and specialized care to achieve this goal. During pregnancy, women with diabetes attend approximately 15 face-to-face visits with health care providers [12]. These include appointments with obstetricians, endocrinologists, diabetes nurses, and dieticians, among others [12]. However, in early pregnancy, a time when the fetus is vulnerable to congenital anomalies [13], less than 15% and 40% of women with type 1 and type 2 diabetes achieve recommended glycemic targets, respectively [14]. Thus, even with intensive and specialized medical care, glycemic control remains suboptimal, contributing to adverse pregnancy outcomes among women with diabetes.

The COVID-19 pandemic has created a barrier to the frequent face-to-face appointments that characterize the medical management of diabetes in pregnancy, highlighting the need for virtual health care. Innovative approaches to virtual health care, such as mobile health (mHealth) technology that facilitates patient-provider communication, offer a promising option to support maternal and fetal well-being. Among nonpregnant adults with diabetes, mHealth interventions are associated with statistically significant and clinically important improvements in glycemic control [15] and there is the potential that mHealth could likewise contribute to improved glycemic control during pregnancy. Although virtual health care modalities, such as mHealth, provide promising options to support the management of chronic conditions, including diabetes in pregnancy, there may also be drawbacks to virtual health care delivery [16]. Marginalized groups in particular, such as patients with language barriers and those who lack access to technology, among others, may face significant challenges [16]. There may also be concerns regarding the quality of virtual health care delivery [16]. Thus, during this time of transition from face-to-face ambulatory care to virtual care, a focus on patient-centered, patient-provider communication is critical. According to King and Hoppe [17], best practice regarding patient-provider communication during medical encounters is communication that contributes to fostering the relationship, gathering information, providing information, making decisions, responding to emotions, and enabling disease- and treatment-related behaviors.

COVID-19 pandemic–induced limitations that impede face-to-face patient-provider communication may compromise the specialized and intensive care that supports expectant mothers with diabetes in achieving glycemic targets and optimizing pregnancy outcomes. It is possible that mHealth interventions that facilitate patient-provider communication may break down barriers and contribute to optimal glycemic control and pregnancy outcomes. These technologies ought to meet best practice standards for patient-centered communication. Therefore, the objective of this scoping review is to map the literature regarding patient-reported benefits and limitations of mHealth technologies that facilitate patient-provider communication in the context of diabetes in pregnancy. We will also determine how the women’s experiences align with the best practice standards for patient-centered communication, as described by King and Hoppe [17].

## Methods

### Study Reporting and Registration

This scoping review protocol was preregistered with Open Science Framework (OSF) on March 25, 2021. Arksey and O’Malley’s framework for conducting scoping reviews [18], with refinements by Levac et al [19] that provide recommendations and clarifications to the original framework, will be used to guide the conduct of this review. According to Arksey and O’Malley, scoping reviews can be conducted to achieve the following: (1) examine the extent, range, and nature of research activity; (2) determine the value for undertaking a full systematic review; (3) summarize and disseminate research findings; and (4) identify research gaps in the existing literature [18]. Scoping reviews allow researchers to incorporate a range of study designs and address questions beyond those related to intervention effectiveness [19]. This scoping review will align with Arksey and O’Malley’s first and fourth scoping review aims. The PRISMA-ScR (Preferred Reporting Items for Systematic Reviews and Meta-Analyses extension for Scoping Reviews) guidelines will guide the reporting of the review [20].

### Identifying the Research Question

The research question is twofold: (1) Among women with diabetes in pregnancy, what are the patient-reported benefits and limitations of mHealth technology? (2) How do the women’s experiences align with the best practice standards for patient-centered communication?

### Identifying Relevant Studies

Relevant studies will be identified by search strategies developed by health science librarians. First, we will search MEDLINE, Embase, Emcare, and PsycINFO for qualitative studies. Secondly, we will search for quantitative literature in MEDLINE, Embase, and Emcare. The reference lists of relevant studies will also be reviewed to identify additional eligible studies. Multimedia Appendix 1 provides the MEDLINE, Embase, Emcare, and PsycINFO search strategy for qualitative studies.

### Study Selection

Following database searches, duplicates will be removed in EndNote and the remaining studies will be transferred to DistillerSR for the title and abstract screening and full-text review. Studies eligible for inclusion are primary studies that report benefits and limitations of mHealth technology used to support or facilitate virtual care for pregnant patients with gestational or pre-existing diabetes. Title and abstract screening will determine whether the study is about mHealth technology in pregnant women with gestational or pre-existing diabetes. The full-text review will determine whether the study elicits patient-reported benefits and/or limitations of mHealth technology. Title and abstract screening and full-text review will be conducted independently by two reviewers (KS and QRW). Any discrepancies will be resolved through discussion or by the inclusion of a third reviewer (DS).

### Charting the Data

Data charting will be completed using a standardized data extraction tool. This tool will first be piloted to ensure accuracy and efficiency during the data charting process. Extracted data will include study characteristics, participant characteristics, and details regarding the described mHealth technologies. All text labelled “results” or “findings” in the included studies will also be extracted. Finally, relevant data will be extracted to determine how the women’s experiences align with King and Hoppe’s best practice standards for patient-centered communication [17]. Data extraction will be conducted by one reviewer (KS) and verified independently by a second reviewer (QRW). Any discrepancies will be resolved through discussion or by the inclusion of a third reviewer (DS).

### Collating, Summarizing, and Reporting the Results

The approach to data synthesis will be adapted from Thomas and Harden’s “Methods for the Thematic Synthesis of Qualitative Research in Systematic Reviews” [21]. This method involves the extraction of all text labelled “results” or “findings” in included studies. The extracted text will be entered verbatim into NVivo. Following the transfer of the text to NVivo, three stages of thematic analysis will be conducted as follows: (1) free line-by-line coding of the study findings; (2) organization of free codes into descriptive themes; and (3) development of analytical themes [21].

## Results

As of August 2020, we have completed the qualitative search strategy. We carried out the qualitative searches in the electronic databases MEDLINE, Embase, Emcare, and PsycINFO (Ovid interface) for a combined total of 8207 articles. Next, we plan to conduct the quantitative searches in the electronic databases MEDLINE, Embase, and Emcare (Ovid interface). We also plan to review the reference lists of relevant studies to identify additional eligible studies. Multimedia Appendix 1 provides the MEDLINE, Embase, Emcare, and PsycINFO (Ovid interface) search strategies for qualitative studies.

## Discussion

For women with diabetes, pregnancy is a critical period that requires intensive and specialized medical management to optimize perinatal outcomes. Among nonpregnant adults with diabetes, mHealth interventions have been shown to improve glycemic control [15]. In the context of COVID-19 pandemic–induced shifts from ambulatory to virtual care delivery, mHealth interventions that enable and support patient-provider communication may potentially serve as a means of improving glycemic control and pregnancy outcomes. However, concerns regarding the quality of virtual health care delivery [16] signal the need for an emphasis on patient-provider communication that is patient-centered. The proposed review will aim to describe the patient-reported benefits and limitations of mHealth technology among women with diabetes in pregnancy and determine how women’s experiences align with the best practice standards for patient-centered communication. The results of this review will be disseminated through peer-reviewed journals and conference presentations to engage relevant stakeholders, including patient-partners, clinicians, researchers and technology developers, and policy makers who are involved in the medical management of women with diabetes in pregnancy.

## Appendix

Multimedia Appendix 1 Qualitative search strategy for Ovid MEDLINE, Embase, Emcare, and PsycINFO.

## Abbreviations

mHealth: mobile health
OSF: Open Science Framework
PRISMA-ScR: Preferred Reporting Items for Systematic Reviews and Meta-Analyses extension for Scoping Reviews
