# Supplementary material for: Patient-Reported Benefits and Limitations of Mobile Health Technologies for Diabetes in Pregnancy: Protocol for a Scoping Review
Source: JMIR Res Protoc. 2021 Oct 29;10(10):e29727. doi: 10.2196/29727 (PMC8590183; doi:10.2196/29727)
Supplement: Multimedia Appendix 1 [file resprot_v10i10e29727_app1.docx]

**Multimedia Appendix 1. Qualitative Search Strategy for Ovid MEDLINE, Embase, Emcare and PsychINFO**

Database: Ovid Medline Epub Ahead of Print, In-Process & Other Non-Indexed Citations, Ovid MEDLINE(R) Daily and Ovid MEDLINE(R) 1946 to Present (2020 October 05)

--------------------------------------------------------------------------------

1 exp diabetes mellitus/ (428758)

2 diabetes.mp. (637169)

3 diabetic.mp. (269389)

4 glucose intolerance.mp. (16472)

5 or/1-4 (716539)

6 exp Pregnancy/ (898081)

7 pregnan$.mp. (1016479)

8 gestation.mp. (121020)

9 (prenatal or antenatal).mp. (197952)

10 or/6-9 (1091939)

11 5 and 10 (40461)

12 (gestation$ adj2 diabet$).tw. (15726)

13 pregnancy-induced diabetes.tw. (11)

14 exp gestational diabetes/ (12777)

15 or/12-14 (19250)

16 11 or 15 (41684)

17 grounded theory/ or exp qualitative research/ (57958)

18 qualitative.tw. (233649)

19 focus group?.tw. (48140)

20 interview$.tw. (366297)

21 experience$.tw. (1102076)

22 (themes or thematic).tw. (92813)

23 hermeneutic?.tw. (3201)

24 grounded theory.tw. (11781)

25 phenomenological.tw. (18308)

26 or/17-25 (1559605)

27 16 and 26 (2382)

Database: Ovid Embase <1974 to 2020 October 05>

--------------------------------------------------------------------------------

1 exp diabetes mellitus/ (967322)

2 diabetes.mp. (1063994)

3 diabetic.mp. (404559)

4 glucose intolerance.mp. (23624)

5 or/1-4 (1161139)

6 exp pregnancy/ (669082)

7 pregnan$.mp. (947101)

8 gestation.mp. (167422)

9 (prenatal or antenatal).mp. (267378)

10 or/6-9 (1095938)

11 5 and 10 (64645)

12 exp pregnancy diabetes mellitus/ (35803)

13 (gestation$ adj2 diabet$).tw. (24828)

14 pregnancy-induced diabetes.tw. (14)

15 or/12-14 (38422)

16 11 or 15 (66399)

17 qualitative research/ or hermeneutics/ (79899)

18 grounded theory/ (7550)

19 focus group?.tw. (60088)

20 interview$.tw. (461618)

21 experience$.tw. (1516046)

22 (themes or thematic).tw. (115186)

23 hermeneutic?.tw. (3539)

24 grounded theory.tw. (14683)

25 phenomenological.tw. (19765)

26 or/17-25 (1940234)

27 16 and 26 (4345)

Database: Ovid Emcare <1995 to 2020 Week 40 (2020 October 05)>

--------------------------------------------------------------------------------

1 exp diabetes mellitus/ (230328)

2 diabetes.mp. (244164)

3 diabetic.mp. (75643)

4 glucose intolerance.mp. (4434)

5 or/1-4 (260600)

6 exp pregnancy/ (124136)

7 pregnan$.mp. (190492)

8 gestation.mp. (35526)

9 (prenatal or antenatal).mp. (62194)

10 or/6-9 (220468)

11 5 and 10 (16410)

12 exp pregnancy diabetes mellitus/ (10568)

13 (gestation$ adj2 diabet$).tw. (6750)

14 pregnancy-induced diabetes.tw. (4)

15 or/12-14 (10981)

16 11 or 15 (16895)

17 qualitative research/ or hermeneutics/ (59474)

18 grounded theory/ (10103)

19 focus group?.tw. (41166)

20 interview$.tw. (244619)

21 experience$.tw. (482566)

22 (themes or thematic).tw. (79070)

23 hermeneutic?.tw. (3002)

24 grounded theory.tw. (11174)

25 phenomenological.tw. (12090)

26 or/17-25 (696896)

27 16 and 26 (1207)

Database: APA PsycInfo <1987 to September Week 4 2020 (2020 October 05)>

--------------------------------------------------------------------------------

1 exp Diabetes/ (17084)

2 diabetes.mp. (29902)

3 diabetic.mp. (6013)

4 glucose intolerance.mp. (444)

5 or/1-4 (31493)

6 exp Pregnancy/ (37635)

7 pregnan$.mp. (58120)

8 gestation.mp. (6613)

9 (prenatal or antenatal).mp. (24206)

10 or/6-9 (76727)

11 5 and 10 (1249)

12 (gestation$ adj2 diabet$).tw. (586)

13 pregnancy-induced diabetes.tw. (0)

14 exp gestational diabetes/ (195)

15 or/12-14 (593)

16 11 or 15 (1300)

17 exp qualitative methods/ or phenomenology/ or qualitative measures/ (28136)

18 exp focus group/ (890)

19 qualitative.tw. (166306)

20 focus group?.tw. (36258)

21 interview$.tw. (307310)

22 experience$.tw. (579799)

23 (themes or thematic).tw. (107537)

24 hermeneutic?.tw. (6324)

25 grounded theory.tw. (16280)

26 phenomenological.tw. (29615)

27 or/17-26 (886615)

28 16 and 27 (273)
